# Magnitude and associated factors of chewing khat during pregnancy in Eastern Ethiopia, 2022: a mixed method cross-sectional study

**DOI:** 10.11604/pamj.2023.46.66.39872

**Published:** 2023-10-24

**Authors:** Amsalu Taye Wondemagegn, Miressa Bekana, Yonas Bekuretsion, Mekbeb Afework

**Affiliations:** 1 Department of Anatomy, School of Medicine, College of Health Sciences, Addis Ababa University, Addis Ababa, Ethiopia,; 2 Department of Biomedical Sciences, School of Medicine, Debre Markos University, Debre Markos, Ethiopia,; 3 Department of Obstetrics and Gynecology, School of Medicine, College of Health Sciences, Haramaya University, Harar, Ethiopia,; 4 Department of Pathology, School of Medicine, College of Health Sciences, Addis Ababa University, Addis Ababa, Ethiopia

**Keywords:** Khat chewing, magnitude, prevalence, associated factors, Ethiopia

## Abstract

**Introduction:**

khat (Catha edulis Forsk), is an evergreen flowering tree or shrub widely produced and consumed in East Africa and the Arabian Peninsula. In Ethiopia, it is largely produced, freely marketed and consumed by almost all segments of the population. This is more pronounced in the Eastern part of the country. However, there exists little scientific information on the level and associated factors of khat use among pregnant women in Ethiopia, despite a few available evidences indicated its adverse effect on pregnancy outcomes. Moreover, the perceived reasons for chewing khat during pregnancy were not yet explored qualitatively to triangulate the quantitative findings. Hence, the aim of the present study was to determine the magnitude and associated factors of chewing khat during pregnancy in Eastern Ethiopia, 2022, in a Mixed Method study approach.

**Methods:**

an institution-based cross-sectional study was conducted from August 1^st^ to 14^th^, 2022 using both quantitative and qualitative methods. Two hundred forty-two pregnant mothers on Antenatal care (ANC) were included in the study. Moreover, 18 purposively selected pregnant mothers on ANC were also considered for qualitative data. Analysis of quantitative data was performed on Statistical Package for the Social Sciences (SPSS) version 27 and thematic framework analysis was performed for qualitative data. Univariable and multivariable logistic regression analysis was performed to identify variables which are significantly associated with khat chewing during pregnancy and to estimate the variable's crude and adjusted odds' ratio with corresponding 95% CI. The statistically significant association was declared at p-value of less than 5%

**Results:**

the overall magnitude of chewing khat during pregnancy in the present study was 27.4% (95% CI: 22.2-33.0). Variables which significantly associated with chewing khat during pregnancy in this study were being >26 years [adjusted odds' ratio (AOR)=2.81 95% CI: 1.19-6.59], being a rural resident (AOR=2.82 95% CI: 1.19-6.69), being illiterate (AOR=4.31 95% CI: 1.02-18.20), participants having chewer husbands (AOR=3.51 95% CI: 1.33-9.24) and respondents having other chewer family members (AOR=3.05 95% CI: 1.19-7.77). In addition, the perceived reasons for chewing khat explored through in-depth interviews were for socialization, to obey tradition, to be happy with friends, husbands and families, to be free from tensioned situations, to be effective in performing daily activities, and due to lack of knowledge of its harm

**Conclusion:**

in the present study, a relatively higher proportion of mothers chewed khat during their current pregnancy. Being older age, living in rural areas, being illiterate, having khat chewer husbands and other family members were statistically significant variables associated with khat chewing during pregnancy. Moreover, pregnant mothers are practicing chewing of khat in the current study area due to deep-rooted sociocultural issues. Hence, creation of awareness on possible harm of chewing khat during pregnancy especially for those found to be at significantly increased risk of consumption in this study is highly recommended during their antenatal care visits. In addition, creation of awareness out of health institutions, at household and community level, is highly recommended, probably using community volunteers (health development armies) in the present study area. At last, we highly also recommend the local government and religious leaders to work on how to stop the chewing practice especially during pregnancy. For example, the local government and religious leaders can work on averting consideration of chewing by the community as culturally accepted practices.

## Introduction

Khat (*Catha edulis* Forsk) is an evergreen flowering tree or shrub which grows mainly in East Africa and the Arabian Peninsula. It belongs to the suborder Rosidae and family Celastraceae. *Catha edulis* forsk, commonly known as “Chat” in Ethiopia but largely mentioned in the literature as khat. It is perceived economically, spiritually and socially as the most important plant in many countries of Eastern Africa, especially in Ethiopia, Somalia, Kenya and Yemen. In Yemen, it has been reported that the prevalence of chewing khat in adult women were found to be 29.6% [1]. In Ethiopia, khat is cultivated in almost all corner of the country on a commercial bases and exported to other countries, specially to neighboring countries. It is also freely marketed and consumed within the country more significantly in Eastern Ethiopia, Harar and Dire Dawa. Based on existing evidences, chewing of khat in Ethiopia is being usual and increasing at an upsetting rate with an estimated prevalence ranged from almost 30% to 53% [2-5]. The highest report being in the Harari region [5]. According to 2016 Ethiopia Demographic and Health Survey (EDHS), about 27% of men and 12% of women had khat chewing practice [6]. Research data on substance use during pregnancy is lacking for most low-and middle-income countries. A study in Yemen, reported 41% prevalence of chewing khat during pregnancy [7]. Documented research evidences of substance use in Ethiopia especially, during pregnancy is limited. According to the existing limited evidences, in addition of adults, the youth and pregnant women are widely chewing khat in Ethiopia. According to recent report, 16.7% [8] of adolescents and 20% [9] of pregnant women in Ethiopia, are practicing chewing khat. In addition, a study done in Eastern Ethiopia showed, 19.6% [10] of pregnant women chew khat. The main quantitatively reported factors in relation to khat chewing in the previous literatures [7,9,10] where age, educational levels, residence, income levels, religion status, health status, partner and family khat chewing status. According to a few available animal experimental studies khat use during pregnancy is associated with adverse maternal and fetal outcomes [11,12]. In the same way, observational studies conducted on human in Yemen [13] and in Ethiopia [14] found a significant association between khat chewing during pregnancy and giving birth of low birth weight infants. Hence, knowing more on the levels and knowing the perceived reasons for chewing during pregnancy in more than one approach is lacking but highly required. This is because it is difficult to quantify perceived reasons (perceptions) but rather could better be explored through qualitative approaches (in this case an in-depth interview). Thus, the aim of the present study was to determine the magnitude and associated factors of chewing khat during pregnancy in Eastern Ethiopia, 2022, in a mixed method study approach. Knowing the current level and understanding the perceived reasons of chewing during pregnancy will enable the policymakers, the health care planners and the health care providers to have evidences and to design local strategies for how to decrease khat chewing during pregnancy.

## Methods

**Study settings and design:** an institution based cross-sectional study was conducted from August 1^st^ to 14^th^, 2022 using both qualitative and quantitative methods of data collection. This preliminary study was conducted in selected hospitals of Eastern parts of Ethiopia (Dire Dawa administration, Harari regional state and Jigjiga city, capital of Somali regional state).

**Sample size and sampling procedure:** sample size was calculated using single population proportion determination formula: by considering proportion of khat chewing (p) 19.6% from previous study [10], 95% confidence interval (α = 0.05), and a margin of error (d) 5%; the final sample size is calculated to be 242. For qualitative data 18 chewer pregnant mothers on antenatal care follow up in the selected hospitals were in depth interviewed. To get study participants, firstly, a total of 4 hospitals, two from Harari regional state, one from Somalia regional state and the other one from Dire Dawa administration was taken. Next, the sampling procedures followed to include and interview the study participants were systematic random sampling techniques. First, the number of pregnant mothers visiting the selected hospitals for antenatal care were observed from the recording offices of the respective selected hospitals. It has been found that on average 25-30 pregnant mothers visited the hospitals ANC units per day. From this, the total number of pregnant mothers who will visit the respective selected hospitals ANC units within 2 weeks of data collection period were determined. The number of study participants to be included and interviewed per day within 2 weeks of data collection period were determined to be 4. Hence, the sampling interval which was 6, is obtained by dividing the total number of pregnant mothers who visited the selected hospitals per day with the number of pregnant mothers interviewed per day. Lastly, every 6^th^ pregnant mothers who had visited the selected hospitals ANC units were included and interviewed during 2 weeks of data collection. For qualitative data, purposive sampling was used to select pregnant mothers on ante-natal care follow-up. Variables studied in the present study were: khat chewing magnitude, age, residence, ethnicity, religion, educational status, occupational status, marital status, household income status, current pregnancy related healthy practices and other substances use history. Healthy practices: in the present study refers to timely initiation and continuity of ANC follow up and restriction from substance use. Perception: in the present study refers to their thinking of why they chew khat.

**Data collection:** data collection tool for interview was developed by reviewing different literatures. The questionnaire was prepared in English and translated in to the respective local languages and back translated in to English to maintain consistency. Data were collected by interviewer-administered questionnaire. The questionnaires are consisting of sociodemographic, pregnancy related, and behavioral characteristics. Health professionals with BSc qualification were used for data collection and supervisions after training by the principal investigators. The training was given by focusing on objective of the study, confidentiality of information, and the contents of the questionnaire in detail. For qualitative data in depth interview of selected pregnant mothers on ANC was conducted using interview guide.

**Data processing and analysis:** each questionnaire data was given a code and entered in to Epi-Data version 3.1 statistical package and exported to SPSS version 27.0 statistical package for statistical analysis. Data cleaning and editing was made before analysis. The result of study is presented in both descriptive statistics (percent, table, mean, median values, dispersion measurements like standard deviation, interquartile range) and inferential statistics (odds ratio). Binary logistic regression was performed to calculate the univariate and multivariate crude and adjusted odds' ratio respectively and to determine independent predictors of the dependent variable. In multi-variate logistic regressions model, only those variables associated with dependent variable with p-value < 0.2 in univariate analysis, and not collinear were entered. Statistical significance was declared at p-value <0.05. Distributions of sociodemographic and behavioral characteristics between khat chewers and non-khat chewer study participants were compared using chi-square tests (Pearson, p-values tested two-sided). Analysis of qualitative data was through thematic framework analysis: 1^st^the interview was typed up or transcribed and was translated into English, then the text was read and key themes were identified. In addition, the responses given was sorted into meaningful categories (groups) by keeping the variations of respondents´ answers in order to aid comparison among respondents. Finally, the qualitative result was presented through narrative form with support of evidences from raw data as direct quotes (paraphrases).

**Data quality management:** to maintain data quality training was given for data collectors and supervisors. Properly designed data collection material was developed by reviewing different literatures. Supervision was carried out on a daily basis to check completeness and consistency by the principal investigators. Correctly complete questionnaires were collected from data collectors and double entered by the principal investigator to check correct data entry. In addition, at the end of data entry data cleaning was done using frequencies, cross tabulations, sorting and listing to check missed values and outliers. Errors identified were corrected by revising the original questionnaire. More importantly, pretest was done using five percent of the sample size on those pregnant women who were not included in the final data collection and errors identified during pre-test were corrected accordingly.

**Ethical considerations:** ethical approval was obtained from Institutional Review Board of College of Health Sciences, Addis Ababa University. Permission was also obtained from the concerned bodies of Harari regional state, Dire Dawa Administration and Somalia region. To protect confidentiality no personal identifier was recorded on the questionnaire and the recorded data was not accessed by a third person. Written informed consent was obtained from study participants to get permission to participate in the study.

## Results

Out of 242 expected study participants, 230 were actually involved in the study, making the response rate of the study 95%. In this study, the mean age of the respondents was 26.7 years with a standard deviation (SD) of 4.69 years. Slightly higher of study participants (51.3%) were living in rural areas. Ethnically, most study participants, 96 (41.7%) were Oromo, followed by Harari, 43 (18.7%) and Amhara, 40 (17.4%). The majority (65.7%) of the study participants were Muslim in religion, followed by orthodox Christians (25.7%). A relatively greater number of study participants, 96 (41.7%) and, 60 (26.1%) were illiterate and farmers in occupation respectively. More than half (74.8%) of involved study participants were married. The median monthly household income of the study participants was 4500 Ethiopian birr with an interquartile range of 2700 Ethiopian birr (Table 1).

**Table 1:** distribution of sociodemographic characteristics by their khat chewing status of the study participants in Eastern parts of Ethiopia, 2022 (N=230)

Variables	Khat chewing practices of the study participants		Total	P-value
	Non chewers, No (%)	Chewers, No (%)		
**Age group of study participants**				
<26 years	82 (83.7%)	16 (16.3%)	98	0.001
≥26 years	85 (64.4%)	47 (35.6%)	132	
Total	167(72.6)	63 (27.4%)	230	
**Participants area of residence**				
Urban	99 (88.4%)	13 (11.6%)	112	<0.001
Rural	68 (57.6%)	50 (42.4%)	118	
Total	167(72.6)	63 (27.4%)	230	
**Participants ethnicity**				
Oromo	63 (65.6%)	33 (34.4%)	96	0.139
Harari	32 (74.4%)	11 (25.6%)	43	
Amhara	35 (87.5%)	5 (12.5%)	40	
Somali	25 (73.5%)	9 (26.5%)	34	
Others (Tigre and Gurage)	12 (70.6%)	5 (29.4%)	17	
Total	167(72.6)	63 (27.4%)	230	
**Participants religion**				
Muslim	105 (69.5%)	46 (30.5)	151	0.347
Orthodox	46 (78%)	13 (22%)	59	
Protestant	16 (80%)	4 (20%)	20	
Total	167(72.6)	63 (27.4%)	230	
**Participants educational status**				
No formal education	61 (63.5%)	35 (36.5%)	96	0.004
Primary education	41 (68.3%)	19 (31.7%)	60	
Secondary education	34 (87.2%)	5 (12.8%)	39	
Tertiary education	31 (88.6%)	4 (11.4%)	35	
Total	167(72.6)	63 (27.4%)	230	
**Participants occupation**				
House wife/homemaker	32 (59.3%)	22 (40.7%)	54	0.012
Farmer	42 (70%)	18 (30%)	60	
Government employee	39 (92.9%)	3 (7.1%)	42	
Non-government employee	9 (81.8%)	2 (18.2%)	11	
Merchant	36 (73.5%)	13 (26.5%)	49	
Daily laborer	9 (64.3%)	5 (35.7%)	14	
Total	167(72.6)	63 (27.4%)	230	
**Participants marital status**				
Currently married	122 (70.9%)	50 (29.1%)	172	0.326
Divorced and widowed	45 (77.6%)	13 (22.4%)	58	
Total	167(72.6)	63 (27.4%)	230	
**Participants monthly HH income (ETB)**				
<4500	72 (69.9%)	31 (30.1%)	103	0.407
≥4500	95 (74.8%)	32 (25.2%)	127	
Total	167(72.6)	63 (27.4%)	230	

HH: household; ETB: Ethiopian birr

**Khat chewing patterns of respondents and their perceived reasons of chewing:** out of 230 respondents, 63 [27.4% (95% CI: 22.2-33.0)] had practiced chewing khat during current pregnancy. Of these, 24 (38.1%) of them chewed daily, 21 (33.3%) chewed more than one day per week and the remaining 18 (28.6%) chewed khat once per a week. Multiple reasons were given for chewing khat among chewers in the current study. Among the reported reasons, the most frequent were: chewing for socialization, 40 (63.5%), to obey tradition, 38 (60.5%), for excited way of life, 31 (49.2%), and unawareness of its harm, 24 (38.1%) (Table 2). These results are also supported by qualitative findings obtained through in-depth interview of pregnant mothers. The major perceived reasons of khat chewing reported by study participants at time of in-depth interview were: to be happy with friends, to be free from tensioned situations, to be effective in performing daily activities, and lack of knowledge of its harm. An in-depth interview of a 28-year-old 9 months pregnant study participant said, *“I have begun to chew khat with my lovely friends. One day I was in a space with my friends which I usually pass my time for recreation. During that time a friend of my friend who had a previous khat chewing experience joined us holding khat and told and encourages us to start chewing to feel free and to be joyful and give us few sticks and then we repeat on the other days. That is how I experienced chewing khat.”* An in-depth interview of another 25-year-old 7 months pregnant respondents also explained, *“I really remembered how I enforced to be khat chewer. When my friends chew khat in a group, I became triggered to join them. I decided to taste it in the first time I started and gradually repeated it for several days in a small amount. Now I become a daily user of khat with large amount. That is how I become a regular khat chewer.”* An in-depth interview of a 30-year-old 6 months pregnant respondent added, “My family are not using khat. I started chewing with my friends while being a high school student. When I was in high school most of my friends were practiced chewing khat and even encourage and enforce others to practice chewing and then I tasted with one stick the first time and repeated it on the consecutive days. Now my eye did not open in the afternoon without chewing.” A 27-year-old, 6 months pregnant respondent added, *“At first time I have begun chewing of khat with my friends, aimed at to try its taste and to know the type of effects it will have on our body and we continue consuming repeatedly being in groups. Now I become daily consumer of khat. Enjoy chewing of khat.”* A 32-year-old, 8 months pregnant respondent reported, *“Life is difficult without khat chewing. Without chewing of khat an individual cannot be able to perform the activities given for him and hence, cannot become competent. In order for me to perform my daily activities effectively chewing of khat is a must. Otherwise, I will lose everything, because I will be enforced to leave my occupation when I cannot perform the activities given for me effectively. So, enjoy chewing of khat.”* A 32-year-old, 7 months pregnant respondent explained, *“I did not believe khat chewing brings any health problems to me and my unborn child. My family and the community hear at large also feels like I said. I have 3 children born before without any health problems in spite of my khat chewing practices and hence, I continued chewing of khat by decreasing its amount from my pre pregnancy amount. This is even due to fear of the healthcare providers´ advice of chewing khat during my current pregnancy will bring problems on my unborn child.”* A 22-year-old 6 months pregnant respondent also added, *“In early period of my pregnancy I was daily consumer of khat. But when I visited health institutions for antenatal care the health care providers told me as my khat chewing practices are dangerous for my unborn child. Then after I stopped chewing due to fear of the problems that will occur on my unborn child.”*

**Table 2:** khat chewing patterns and perceived reasons of chewing of pregnant mothers in Eastern parts of Ethiopia, 2022 (N=230)

Variables	Response choice	Frequency	Percent
Khat chewing during current pregnancy (n=230)	Yes	63	27.4%
	No	167	72.6%
Frequency of khat chewing (n=63)	Daily	24	38.1%
	More than one day per week	21	33.3%
	Once per week	18	28.6%
Reasons of chewing khat of the respondents (n=63)	For socialization	40	63.5%
	For obeying tradition	38	60.5%
	For excited way of life	31	49.2%
	Unaware of its harm	24	38.1%
	For coping up partner pressure	19	30.2%
	For coping up peer pressure	18	28.6%
	For coping up family pressure	16	25.4%
	For managing life pressure	7	11.1%

**Khat chewing related behaviors and current pregnancy related healthy practices of respondents:** among the total study participants, only 23 (10%) had reported alcohol use during current pregnancy with a relatively higher proportion (17.5%) among chewers as compared to non-chewers, 7.2%. Similarly, only 15 (6.5%) of study participants reported tobacco smoking during current pregnancy with comparable proportion among chewers and non-chewer study participants. Almost half, 110 (47.8%) and, 111 (48.3%) of study participants respectively had husbands and other family members who practiced khat chewing. All chewer study participants gestational age at time of conducting the study were 8 and 9 months with higher proportion of 9 months. On the other hand, non-chewer study participant´s gestational ages at time of interview were between 5 and 9 months. A higher proportion (74.6%) of chewers in the present study had one ANC visits at time of interview (Table 3).

**Table 3:** comparison of khat chewing related behaviors and current pregnancy related healthy practices of respondents in Eastern parts of Ethiopia, 2022 (N=230)

Variables	Khat chewing practices of study participants		Total	P-value
	Non chewers, No (%)	Chewers, No (%)		
**Alcohol use during current pregnancy**				
Yes	12 (52.2%)	11 (47.8%)	23	0.021
No	155 (74.9%)	52 (25.1%)	207	
Total	167 (72.6)	63 (27.4%)	230	
**Cigarrete smoke during current pregnancy**				
Yes	10 (66.7%)	5 (33.3%)	15	0.59
No	157 (73%)	58 (27%)	215	
Total	167(72.6)	63 (27.4%)	230	
**Husbands’ khat use**				
Yes	60 (54.5%)	50 (45.5%)	110	<0.001
No	107 (89.2%)	13 (10.8%)	120	
Total	167(72.6)	63 (27.4%)	230	
**Other family members khat use**				
Yes	61 (55%)	50 (45%)	111	<0.001
No	106 (89.1%)	13 (10.9%)	119	
Total	167 (72.6)	63 (27.4%)	230	
**Gestational age at time of interview**				
5 months	6 (100%)	0	6	<0.001
7 months	37 (100%)	0	37	
8 months	46 (66.7%)	23 (33.3%)	69	
9 months	78 (66.1%)	40 (33.9%)	118	
Total	167(72.6)	63 (27.4%)	230	
**Antenatal care visits number at time of interview**				
1	27 (36.5%)	47 (63.5%)	74	<0.001
2	14 (63.6%)	8 (36.4%)	22	
3	43 (93.5%)	3 (6.5%)	46	
4	83 (94.3%)	5 (5.7%)	88	
Total	167(72.6)	63 (27.4%)	230	
**Is current pregnancy planned?**				
Yes	153 (74.3%)	53 (25.7%)	206	0.098
No	14 (58.3%)	10 (41.7%)	24	
Total	167(72.6)	63 (27.4%)	230	

**Factors associated with chewing khat during current pregnancy:** in the present study, binary logistic regression analysis showed: age, area of residence, educational level, occupation, alcohol use, partners khat chewing and other family members khat chewing were among the factors which significantly associated with current khat chewing of study participants. Of these factors, occupation and alcohol use did not show significant association with current khat chewing on multivariable logistic regression analysis. In this study, participants aged 26 and above years had a 2.81 times higher (AOR=2.81 95% CI: 1.19-6.59) risk of khat chewing practices during their current pregnancy as compared to those participants aged less than 26 years. Participants living in rural area had 2.82 times increased (AOR=2.82 95% CI 1.19-6.69) risk of khat chewing as compared to those study participants living in urban area. Illiterate study participants were 4.31 times at higher risk of khat chewing compared to those participants with tertiary education level (AOR=4.31 95% CI: 1.02-18.20). Participants having chewer husbands were 3.51 times at higher (AOR=3.51 95% CI: 1.33-9.24) risk of chewing khat as compared to those respondents having no chewer husbands. In the current study, respondents with other family members who practiced khat chewing were 3.1 times (AOR=3.05 95% CI: 1.19-7.77) at increased risk of khat chewing during current pregnancy as compared to those study participants having no other chewer family members (Table 4). There was no identified multicollinearity among independent variables [variance inflation factor (VIF)<5) and tolerance >0.1].

**Table 4:** bivariable and multivariable logistic regression of chewing khat during current pregnancy in Eastern Ethiopia, 2022 (N=230)

Variables	Khat chewing practices of study participants		COR (95% CI)	AOR (95% CI)
	Non chewers, No (%)	Chewers, No (%)		
**Age group of study participants**				
<26 years	82 (83.7%)	16 (16.3%)	1	1
≥26 years	85 (64.4%)	47 (35.6%)	2.83 (1.49-5.39)**	2.81 (1.19-6.59)*
**Participants area of residence**				
Urban	99 (88.4%)	13 (11.6%)	1	1
Rural	68 (57.6%)	50 (42.4%)	5.6 (2.83-11.09)***	2.82 (1.19-6.69)*
**Participants ethnicity**				
Oromo	63 (65.6%)	33 (34.4%)	1.26 (0.41-3.87)	1.60 (0.33-7.69)
Harari	32 (74.4%)	11 (25.6%)	0.83 (0.24-2.87)	1.71 (0.31-9.32)
Amhara	35 (87.5%)	5 (12.5%)	0.34 (0.08-1.39)	0.98 (0.15-6.37)
Somali	25 (73.5%)	9 (26.5%)	0.86 (0.24-3.15)	1.02 (0.17-6.04)
Others (Tigre and Gurage)	12 (70.6%)	5 (29.4%)	1	1
**Participants religion**				
Muslim	105 (69.5%)	46 (30.5)	1.75 (0.56-5.53)	0.85 (0.19-3.71)
Orthodox	46 (78%)	13 (22%)	1.13 (0.32-3.97)	0.95 (0.18-5.07)
Protestant	16 (80%)	4 (20%)	1	1
**Participants educational status**				
No formal education	61 (63.5%)	35 (36.5%)	4.45 (1.45-13.64)**	4.31 (1.02-18.20)*
Primary education	41 (68.3%)	19 (31.7%)	3.59 (1.11-11.63)*	2.43 (0.53-11.13)
Secondary education	34 (87.2%)	5 (12.8%)	1.14 (0.28-4.63)	0.75 (0.13-4.38)
Tertiary education	31 (88.6%)	4 (11.4%)	1	1
**Participants occupation**				
House wife/homemaker	32 (59.3%)	22 (40.7%)	1	1
Farmer	42 (70%)	18 (30%)	0.62 (0.29-1.35)	0.52 (0.19-1.39)
Government employee	39 (92.9%)	3 (7.1%)	0.11 (0.03-0.41)**	0.37 (0.07-2.04)
Non-government employee	9 (81.8%)	2 (18.2%)	0.32 (0.06-1.64)	1.06 (0.12-9.47)
Merchant	36 (73.5%)	13 (26.5%)	0.53 (0.23-1.21)	0.83 (0.27-2.52)
Daily laborer	9 (64.3%)	5 (35.7%)	0.81 (0.24-2.74)	1.38 (0.26-7.34)
**Participants marital status**				
Currently married	122 (70.9%)	50 (29.1%)	1.42 (0.71-2.86)	2.11 (0.82-5.39)
Divorced and widowed	45 (77.6%)	13 (22.4%)	1	1
**Participants monthly household income (ETB)**				
<4500	72 (69.9%)	31 (30.1%)	1	1
≥4500	95 (74.8%)	32 (25.2%)	0.78 (0.44-1.39)	1.58 (0.68-3.68)
**Alcohol use during current pregnancy**				
Yes	12 (52.2%)	11 (47.8%)	2.73 (1.14-6.56)*	2.28 (0.64-8.18)
No	155 (74.9%)	52 (25.1%)	1	1
**Cigarette smoke during current pregnancy**				
Yes	10 (66.7%)	5 (33.3%)	1.35 (0.44-4.13)	2.06 (0.44-9.65)
No	157 (73%)	58 (27%)	1	1
**Husbands’ khat use**				
Yes	60 (54.5%)	50 (45.5%)	6.86 (3.45-13.64)***	3.51 (1.33-9.24)*
No	107 (89.2%)	13 (10.8%)	1	1
**Other family members khat use**				
Yes	61 (55%)	50 (45%)	6.68 (3.36-13.28)***	3.05 (1.19-7.77)*
No	106 (89.1%)	13 (10.9%)	1	1

## Discussion

The magnitude of chewing khat among pregnant mothers in the present study was 27.4%. This magnitude is lower than studies conducted in Yemen, which reported 41% magnitude of chewing khat during pregnancy [7] and 29.6% prevalence of khat chewing in adult women [1]. The variability may be attributed to study area, study population and sociocultural variability. Similarly the current finding is lower than local studies: a study in Jimma reported 37.8% magnitude of khat chewing [15] in general populations, a study in Harar reported 53% prevalence of khat chewing [5] in general populations, and studies in Butajira, Ethiopia found 50% prevalence of chewing khat [16] in general population and 35.8% prevalence of khat chewing during pregnancy [17]. The variability may be due to the differences in the study area, study period, study populations and approach. In most areas of Ethiopia female participation in khat chewing practices are low as compared to males [18] due to cultural taboo and restrictions. A previous study reported that 27% of men and 12% of females in Ethiopia are khat chewers [6]. But in some areas of Ethiopia females´ involvement in khat chewing practices is not culturally and socially prohibited. On the other hand, the present magnitude of chewing khat during pregnancy is higher than previous local studies which reported 20% prevalence in a systematic review and meta-analysis in Ethiopia [9], 19.6% magnitude [10] and 9.9% prevalence of chewing during pregnancy [19]. This variability may be attributed to sociocultural variations. Even though the participation of females in khat chewing practices in most parts of Ethiopia are limited due to sociocultural taboo, the involvement of women in khat chewing in Harar, Eastern Ethiopia is culturally as well as socially accepted practice as seen in the current study and this finding is in agreement with a previous report [20]. Hence, the study participants may freely report their chewing practice at time of interview and there is a less chance of underreporting of their chewing practices. In the present study a higher proportion of study participants were chewing khat for socialization, and to obey tradition. These findings can also be supported by the qualitative findings obtained thorough an in-depth interview of pregnant mothers. A 27-year-old 7 months pregnant study participant at in depth interview reported, *“Chewing khat hear in Harar is a common traditional daily practice of the population which I have seen in my day-to-day life. As part of this community, I consumed khat daily because I received it from the population and I fill proud of my chewing and hence, I can say chewing khat in Harar is our culture.”*

Another 27-year-old 6 months pregnant study participants explained, *“Being living in Harar, it is impossible to live without chewing. Chewing khat is highly valued traditional practices in the community, in which the chewing practices are transmitted from elders to our generation. Hence, I have consumed khat because it is my community and as well my families´ cultural practices.”* Moreover, 32-year-old 8 months pregnant mother also explained the condition in Harar as follows: *“Life in Harar is unique. Because every individual whether rich or poor, older or young, male or female all are participated in khat chewing practices, preferably in groups. Everybody is buying his/her own khat and come together to chew and to discussion different issues related to the community. Hence, in order to participate in the discussion of community issues and to live together in the community chewing of khat is a must. Otherwise, you are not counted as part of the community. The majority of the community sees you, as you are not accepting the culture and tradition of the community. Hence, I used khat for many years.”* In the present study participants with relatively older age, being living in rural area, being illiterate, having chewer husbands and other family members were significantly consumed khat during their current pregnancy. Participants aged 26 and above years were a significantly increased risk of chewing khat during their current pregnancy as compared to those aged less than 26 years. The finding is consistent with previous studies [1,7,21], which reported statistically significant khat chewers were aged between 25 and 49 years. This could be due to the fact that young mothers are more likely to depend on others for their livelihood and may be in lack of income which may greatly decrease their involvement in khat chewing. In addition, older age mothers may have gradually developed the habit through the years. In our study, 54 (23.5%) of the study participants were housewife or home makers. Of this, 37 (16.1%) of them were aged 26 and above years. In addition, young mothers may be under control of their family that may decrease their involvement in khat chewing practices. More importantly, younger women may fear the harm of khat chewing on unborn child as compared to older mothers, as older mothers may have previous children born healthy.

In the present study participants living in rural area were at significant risk of chewing khat during pregnancy compared to those living in urban areas. This result is in line with previous studies [5,7,22]. This may be due to the fact that participants living in rural area may be in lack of access to healthcare services to be advised on healthy practices as compared to urban residents. It has been highlighted that access to health services was variable in rural and urban areas in Ethiopia [23]. In addition, rural residents could lack means to get information about healthy practices as compared to urban residents. More importantly, since khat is mostly farmed in rural areas, the study participants in rural area are more accessible to the product. Lack of education of study participants were at significantly increased risk of khat chewing during pregnancy compared to educated study participants. The finding is consistent with previous study findings [7,9,24]. The possible justification for this could be due to the fact that illiterate mothers may be in lack of information and knowledge about the negative health impacts of khat chewing on unborn child and mothers compared to educated mothers. In addition, health seeking behaviors of educated mothers may be greater as compared to illiterate mothers and may visit the health institutions and hence, may be advised on the harm of chewing khat on themselves and on their unborn child. In the present study out of the total 74 study participants having only one ANC visit at 7 and greater months of gestation, 39 (52.7%) were illiterate. On the other hand, out of the total 88 study participants having 4 ANC visits at 7 and greater months of gestation, 62 (70.5%) were having primary and above levels of education. A previous study also reported literate mothers are more likely utilized appropriate antenatal care than illiterate mothers [25]. Participants having khat chewer husbands in the present study were at significantly increased risk of chewing khat during current pregnancy as compared to those participants having no chewer husbands. The result is in line with previous studies conducted elsewhere [1,9,10,19,22]. The possible justification for the finding could be khat chewer husbands´ may encourage and influence their wives to involve in khat chewing practices and hence, women may share the practice. In the present study 30.2% of the study participants chewed khat to cope up with their partner´s pressure. A qualitative finding obtained through in-depth interview of pregnant women also strengthens this finding. A 34-year-old 9 months pregnant respondent explained the role of her husband in her khat chewing practices as follows, *“I was primarily not in Harar. I was born in an area and family where chewing of khat is highly forbidden. I came to Harar due to marriage when my current husband joined the area as government employee. Then after, we displaced from my birth area to Harar. When I joined Harar my husband encourages and enforces me to start chewing khat to be part of the community and hence, I begun chewing few sticks with sugar and now I become regular chewer of khat.”*

Another 29-year-old 6 months pregnant study participants also explained the role of her husband in her khat chewing practices as follows, *“In my khat chewing experience my husband plays a great role. At first, I fear khat chewing since I saw my neighbor elder man became manic which I think is related to his long-time chewing practices of khat. But my husband reassured me, as the case of our neighbor man was related to his family rather than his chewing practices. And told and repeatedly encourages me to start and enjoy chewing of khat to be healthy thinker and to have joyful family. Now I enjoyed chewing of khat.”* In the current study respondents with khat chewer family members were at significantly increased risk of chewing khat during pregnancy as compared to those having no chewer family members. The present finding is in agreement with previous studies conducted elsewhere [10,21,22]. This could be due to the fact that participants living with chewer family members will be more likely to be influenced by their chewing behaviors. In this study 25.4% of study participants chewed khat to cope up with their family pressures. This result is also supported by qualitative findings obtained through an in-depth interview of pregnant mothers. An in-depth interview of 27-year-old pregnant respondents said, *“I practiced chewing khat since almost all except children of my family members are khat chewers. Without chewing khat I feel I will not be part of the family members and not to be alone I begun chewing khat. Now I am daily consumer of khat.”* On an in depth interview a 32-year-old 7 months pregnant study participant added, *“In our family our elders are practiced khat chewing being in groups. They encouraged us to begin chewing khat by providing small amount and also told us on how to use it the first time. Now I enjoyed chewing khat. That is how I begun chewing khat.”* The cross-sectional nature of the present study did not reveal temporal relationships between the dependent and independent variables. But being a multi-centered study and conducted using both quantitative and qualitative methods are the major strengths of the present study.

## Conclusion

In the present study a relatively higher proportion of mothers were chewed khat during their current pregnancy. Bing older age, living in a rural area, being illiterate, having khat chewer husbands and other family members were statistically significant variables associated with khat chewing during pregnancy. Hence, creation of awareness on possible harm of chewing khat during pregnancy especially for those found to be at significantly increased risk of consumption in this study is highly recommended during their antenatal care visits. In addition, creation of awareness out of health institutions, at household and community level is highly recommended probably using community volunteers (health development armies) in the present study area. At last, we highly also recommend the local government leaders and religious leaders to work on how to stop the chewing practice during pregnancy. For example, the local government and religious leaders can work on averting consideration of chewing by the community as culturally accepted practices

### What is known about this topic

- *The khat chewing practice during pregnancy was quantitatively reported in the previous literature along with other substances*.

### What this study adds

- *Revealing the current magnitude and associated factors of khat chewing during pregnancy in the study area which was exclusively conducted on khat consumption rather than in addition of other substances in which the previous study was conducted;*
- *Qualitative measurement of the perceived reasons of pregnant mother for consuming khat was performed only in the present study*.
